# Cohesion mechanisms for bioadhesives

**DOI:** 10.1016/j.bioactmat.2021.11.008

**Published:** 2021-11-11

**Authors:** Yazhong Bu, Abhay Pandit

**Affiliations:** a Institute of Medical Engineering, Department of Biophysics, School of Basic Medical Sciences, Health Science Center, Xi'an Jiaotong University, Xi'an, 710061, China; b CÚRAM, SFI Research Centre for Medical Devices National University of Ireland, Galway, Ireland

**Keywords:** Bioadhesive, Cohesion, Hydrogel, Medical device fixation, Sealant

## Abstract

Due to the nature of non-invasive wound closure, the ability to close different forms of leaks, and the potential to immobilize various devices, bioadhesives are altering clinical practices. As one of the vital factors, bioadhesives' strength is determined by adhesion and cohesion mechanisms. As well as being essential for adhesion strength, the cohesion mechanism also influences their bulk functions and the way the adhesives can be applied. Although there are many published reports on various adhesion mechanisms, cohesion mechanisms have rarely been addressed. In this review, we have summarized the most used cohesion mechanisms. Furthermore, the relationship of cohesion strategies and adhesion strategies has been discussed, including employing the same functional groups harnessed for adhesion, using combinational approaches, and exploiting different strategies for cohesion mechanism. By providing a comprehensive insight into cohesion strategies, the paper has been integrated to offer a roadmap to facilitate the commercialization of bioadhesives.

## Highlights

• Bioadhesive are altering clinical practices.
• Bioadhesives for medical applications needs different cohesion strategies.
• Better understanding of cohesion mechanism can design suitable bioadhesives.

## Graphical abstract

**Figure:**
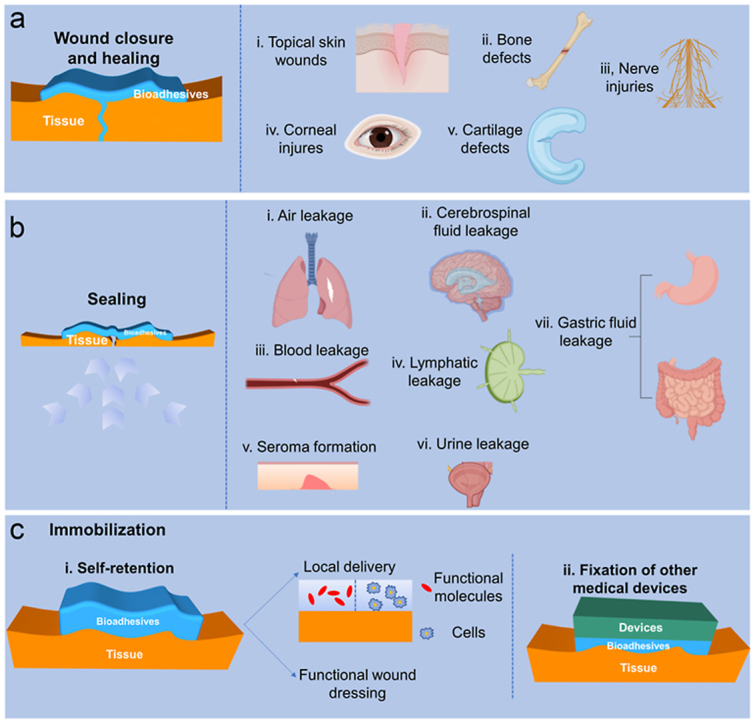

## Abbreviations

DOPA: Dopamine
FGA: Functional groups for adhesion
HRP: Horseradish peroxidase
PEG: Poly(oxyethylene)
PPG: Poly(propylene glycol)
NHS: *N*-Hydroxysuccinimide
NHS-ester: *N*-Hydroxysuccinimide activated ester
PF127: Pluronic®F127
PDA: Polydextran
PEI: Ployethylenimine
PEO: Polyethylene oxide
PVA-A: Polyvinyl alcohol co-vinylamine
HA: Hyaluronic acid
COOH: Carboxyl groups
EDC: 1-Ethyl-3-(3-dimethylaminopropyl)carbodiimide
FDA: Food and Drug Administration
DA: Diels-Alder
AAC: Azide and Alkyne cycloaddition
HEPES: 4-(2-Hydroxyethyl)-1-piperazineethanesulfonic acid
TZ/TCO: Tetrazine/*trans*-Cyclooctene
ADH: Adipic dihydrazide
NB: *N*-(2-aminoethyl)−4-(4-(hydroxymethyl)−2-methoxy-5-nitrosophenoxy)
PEO-PPO-PEO: Poly(ethyleneoxide)-poly(propylene oxide)-poly(ethylene oxide)
PNIPAAm: Poly(*N*-isopropylacrylamide)
FeCl_3_: Iron(III) chloride
NaOH: Sodium hydroxide
H_2_O_2_: Hydrogen peroxide
AgNO_3_: Silver nitrate
NaIO_4_: Sodium periodate
MgO: Magnesium oxide
KMnO_4_: Potassium permanganate
Mfps: mussel foot proteins
ROS: reactive oxygen species
PNIPAm: Poly(*N*-isopropyl acrylamide)
MTT: 3-(4,5-Dimethylthiazol-2-yl)-2,5-Diphenyltetrazolium Bromide
MTS: 3-(4,5-Dimethylthiazol-2-yl)-5-(3-carboxymethoxyphenyl)-2-(4-sulfophenyl)-2H-tetrazolium

## 1 Introduction

**Fig. 1:**
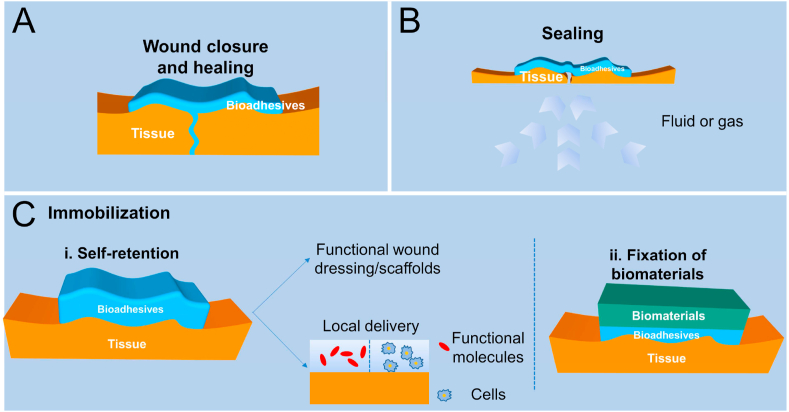
Potential applications of bioadhesives. A, Use of bioadhesives for wound closure. B, Use of bioadhesives as sealants to prevent different internal leakage, including fluid and gas leakage. C, Use of bioadhesives to immobilize separate components. (i) Self-retention. They can act as a delivery system to realize local delivery of functional molecules and cells for long retention. These can also be retained at the application site to serve as functional wound dressings. (ii) As regular adhesives that can be applied to bond various items together, bioadhesives can also be used to fix other medical devices on tissues.

Bioadhesive, extended from ‘adhesive’, can be broadly defined as any substance with characteristics that allow for polymerization, holding either tissues or tissues with other substrates together [1,2]. They are revolutionizing the surgical process. In wound closure, compared to conventional invasive procedures, including suture, staples and wires, bioadhesives cause less pain with a better result, and are preferred by both patients and clinicians.[[3], [4], [5]] They have also been successfully used in leakage prevention with many Food and Drug Administration (FDA) approved products, including Tisseel®, Coseal®, Duraseal®, Progel™, etc. More importantly, because of the ability to immobilize both themselves and other substrates on-site, the applications of bioadhesives are far more than just wound closure and sealing, and are being widely explored in applications such as functional wound dressings, drug/cell delivery carriers and fixation of biomaterials/tissue scaffolds (Fig. 1) [[6], [7], [8], [9], [10], [11], [12], [13]].

It is known that the adhesion performance of the adhesives is determined by two different forces: adhesion and cohesion [1,2,14]. Adhesion refers to the intermolecular forces maintaining the bond between the adhesives and the adherent tissue surface. In contrast, cohesion refers to the internal strength of the adhesive for holding the network together [15,16]. As with adhesion, cohesion also counts for adhesion strength. Failure in bioadhesives is sometimes a result of cohesive failure due to their poor mechanical stability [17]. Dopamine (DOPA)-inspired polymers typically suffer from inadequate cohesive strength, resulting in easily deformed or stretched detachment from adhered surfaces [18,19]. More importantly, cohesion directly determines the translation of bioadhesives. Different clinical scenarios need different formulations of bioadhesives for ease of use. For a healing wound with large volumes, an injectable bioadhesive that can adhere the deep wounds together is preferable to the adhesive patch. Injectable bioadhesives are also advantageous in treating irregular injuries. For stopping the bleeding with continuous blood flow, a preformed adhesive patch is more useful than injectable ones because it will not be easily washed away, and outside pressure can be used applied directly for better efficacy. To be applied on the surface of a beating heart, a sprayable bioadhesive will be more appropriate [20]. All these formulations can be achieved by purposely designing the cohesion mechanisms of bioadhesives. For example, Oxidation of DOPA is commonly used to fabricate *in situ* injectable bioadhesives, while photo-crosslinking is an excellent way to develop an adhesive patch [6,8,18]. Ease of use is always an important factor for medical devices' translation, so by determining the method of application, cohesion is an important factor determining bioadhesives' translation.

Moreover, cohesion can endow different smart functions to bioadhesives. Functions like thermo-responsiveness for better injectability, anti-water for being stable in highly humid environment within the body, controllable dissolution for non-invasive removal of bioadhesives, and self-healing properties in case of wound dehiscence can be achieved using different chemical/physical strategies in cohesion design [[21], [22], [23], [24], [25], [26]]. When bioadhesives are used as wound dressings, the way to change them when needed should be considered and smartly removable property will simplify this process [27]. To solve this problem, thiol-Thioester exchange has been introduced into the network for controllable dissolution to allow for re-exposure of wounds after applying adhesives [28]. Because of dynamic equilibrium between the Schiff base and aldehyde and amine reactants in aqueous solutions, the Schiff base reaction has been used to fabricate bioadhesives with a self-healing cohesion property [29]. By choosing the right cohesion strategies, bioadhesives with proper adhesion strength with different functions for desired applications can be fabricated [6,8].

However, although the adhesion mechanisms are reasonably well discussed in many reviews [15,[30], [31], [32], [33]] there are very few reviews that discuss the mechanisms for cohesion. Hence, in this review, we have summarized the most common methods in cohesion strategies. Discussions about the influences of those mechanisms on the performance of the bioadhesives are presented. Further, to offer a whole map for bioadhesive fabrication, the relationship between the cohesion and adhesion mechanisms is also discussed, including using the same functional groups, using extra functional groups for cohesion, and using totally different functional groups for cohesion [1,2,31,32,[34], [35], [36]]. It is hoped that by integrating the cohesion mechanisms, this review can facilitate the translation of bioadhesives.

## 2 Cohesion strategies

**Fig. 2:**
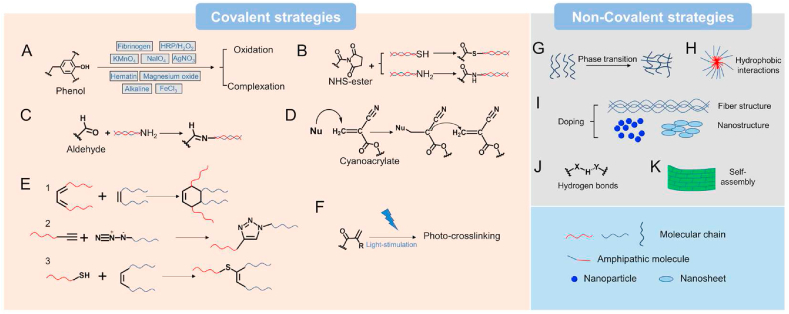
**The common strategies for cohesion.** The cohesion strategies include covalent (A to F) and non-covalent (G to K) ones. They are (A) phenol groups-based strategy, (B) NHS-ester-based strategy, (C) aldehyde groups-based strategy, (D) cyanoacrylate-derivatives-based strategy, (E) click chemistry-based strategy including Diels-Alder (DA) reactions, Azide and Alkyne cycloaddition (AAC) reactions, and Thiol-ene reactions, (F) photo-crosslinking-based strategy, (G) phase transition-based strategy, (H) hydrophobic interactions-based strategy, (I) doping-based strategy, (J) hydrogen bonds-based strategy and (K) self-assembly-based strategy.

There are plenty of strategies for cohesion, ranging from covalent strategies to non-covalent strategies. In this article, to maximize the conciseness, only the most commonly used strategies have been listed, which have been illustrated in Fig. 2 and Table 1. According to the nature of strategies, we have grouped them into two major categories, including covalent (Fig. 2A–F) and non-covalent ones (Fig. 2G–K). For covalent strategies, phenol groups, *N*-Hydroxysuccinimide activated ester (NHS-ester), aldehyde, cyanoacrylate, click reactions and photo-crosslinking-based ones are the most common ones. In non-covalent strategies, phase transition, hydrophobic interactions, doping, hydrogen bonds and self-assembly-based ones have been explored. The detailed discussion about those strategies is presented as follows.

**Table 1:** Commonly used cohesion strategies as well as some examples.

Materials used	Cohesion mechanism	Adhesion mechanism	Applications	Ref
**Covalent strategies-Phenol groups-based strategy**				
Albumin, DOPA, Citrate acid	FeCl_3_ and NaOH crosslinked DOPA	DOPA	Seroma prevention	[44]
DOPA modified hyaluronic acid and reduced graphene oxide	H_2_O_2_ and HRP crosslinked DOPA	DOPA	Hemostasis, functional wound dressing	[45]
Mussel adhesive protein, hyaluronic acid	NaIO_4_ crosslinked DOPA	DOPA	Urine leakage	[153]
Citric acid, PEG-PPG-PEG, DOPA, magnesium oxide	Magnesium oxide crosslinked DOPA	DOPA	Wound closure	[154]
DOPA modified carboxymethyl cellulose	H_2_O_2_ and HRP crosslinked DOPA	DOPA	Wound closure/dressing	[155]
Thiol and catechol-conjugated chitosan	NaIO_4_ crosslinked catechol	Catechol	Wound closure	[156]
DOPA modified poly(α,β-aspartic acid) derivative	FeCl_3_ crosslinked DOPA	DOPA	Drug delivery	[157]
DOPA modified chondroitin sulfate	FeCl_3_ crosslinked DOPA	DOPA	Seroma prevention/hemostasis	[158]
Thiourea and DOPA modified Gelatin	H_2_O_2_ and HRP crosslinked DOPA	DOPA	Cell delivery	[159]
Tannic acid modified gelatin	Oxidation of polyphenol groups	Oxidation of polyphenol groups	N/A	[51]
HA, gelatin, tyrosinase	Tyrosinase crosslinking	Tyramine	Tissue engineering and regenerative medicine	[37]
Epigallocatechin gallates and tyramine conjugated hyaluronic acids	Oxidation of phenol groups	Oxidation of phenol groups	Wound closure	[38]
**Covalent strategies-NHS-ester-based cohesion**				
NHS terminated PEG, peptide dendrimer	With amino terminated peptide dendrimer	Active ester	Sealing	[28,59]
Gelatin, NHS terminated PEG	With gelatin	Active ester	Sealing	[58]
Gelatin, NHS terminated PEG	With Gelatin	Active ester	Sealing	[65]
NHS terminated PEG, lysozyme	With lysozyme	Active ester	Sealing	[57]
Gelatin (coldwater fish) and alginate	With Gelatin	COOH with EDC	Sealing	[66]
Amino and NHS modified PEG	With amino terminated PEG	Active ester	Wound closure/sealing/drug delivery	[62]
Gelatin, alginate	With Gelatin	COOH with EDC/NHS	N/A	[160]
**Aldehyde-based cohesion**				
Quaternized Chitosan, benzaldehyde modified PF127	With chitosan	Aldehyde	Wound dressing	[29]
Aldehyde modified dextran, Chitosan	With chitosan	Aldehyde	Sealing	[70]
Aldehyde and amine terminated PEOs	With amine terminated PEOs	Aldehyde	N/A	[161]
Carboxymethyl chitosan, gelatin, Aldehyde modified alginate	With chitosan and gelatin	Aldehyde	Drug delivery and hemostasis	[68]
Aldehyde modified alginate, gelatin	With gelatin	Aldehyde	Wound closure	[80]
Benzaldehyde functionalized PEG, Quaternized Chitosan	With chitosan	Aldehyde	Wound dressing	[143]
**Covalent strategies-Cyanoacrylate-based cohesion**				
2-octyl cyanoacrylate	2-octyl cyanoacrylate	2-octyl cyanoacrylate	Wound closure	[88]
DOPA, allyl 2-cyanoacrylate	DOPA, allyl 2-cyanoacrylate	DOPA, allyl 2-cyanoacrylate	Wound closure	[89]
PLCL modified allyl 2-cyanoacrylate	PLCL modified allyl 2-cyanoacrylate	PLCL modified allyl 2-cyanoacrylate	Wound closure	[162]
**Covalent strategies-Click chemistry**				
PEG, citric acid, DOPA, gelatin	Click chemistry enhancement with NaIO_4_ crosslinked DOPA	DOPA	Wound closure	[18]
Aldehyde modified PEG, cyclooctene (TCO)/tetrazine (Tz) modified chitosan	Schiff base crosslinking with click chemistry	Aldehyde	Cartilage regeneration	[94]
Furylamine, dihydrazide and aldehyde modifed hyaluronic acid, Dimaleimide PEG	Schiff base crosslinking and click chemistry	Aldehyde	Wound dressing and immobilization	[79]
**Covalent strategies-Photo-crosslinking**				
Laponite, PEG, DOPA	Nanocomposite enhancement with DOPA oxidation	DOPA	Sealing	[115]
Maleic anhydride modified chitosan, benzaldehyde modified PEG, PEG diacrylate, methacrylamide modified DOPA	Photocrosslinking and Schiff base crosslinking	DOPA and aldehyde	Sealing	[127]
Gelatin, glycosaminoglycan hyaluronic acid	Photocrosslinking and Schiff base crosslinking	Aldehyde	Sealing/Wound dressing	[103]
Aldehyde and methacrylate modified alginate, Amino terminated PEG	Schiff base crosslinking and photocrosslinking	Aldehyde	Drug delivery, wound closure, wound dressing, immobilization of medical devices	[74]
**Non-covalent strategies**				
Poly(lactic-*co*-glycolic acid) (PLGA), poly(ethylene glycol) (PEG), nano-tomicroscale and silica particles	Silica particles together with the polymer blend	Mechanical Interlocking	Sealing and hemostasis	[163]
Acrylate modified DOPA, Acrylamide	Hydrophobic association with FeCl_3_ crosslinked DOPA	DOPA		[109]
DOPA modified hyaluronic acid, thiol modified Pluronic F127 copolymer	Michael addition between DOPA and thiol, and phase transition	DOPA	Drug or cell delivery	[21]
Hydrocaffeic acid modified Chitosan, thiol modified Pluronic F-127	Michael addition between catechol and thiol, and phase transition	Catechol	Wound closure/hemostasis	[22]
Poly (*N*-isopropylacrylamide) grafted chondroitin sulfate, aldehyde modified chondroitin sulfate, liposomes	Schiff base crosslinking and phase transition	Aldehyde	Tissue engineering	[78]
Hydrazide-modified poly(l-glutamic acid), catechol- and aldehyde-modified alginate	Schiff base	Catechol and aldehyde	Wound closure and hemostasis	[23]
Alginate aldehyde, borate, gelatin	Schiff base crosslinking with borate complexation	Aldehyde	Adhesion/tissue engineering	[69]

### 2.1 Covalent strategies

#### 2.1.1 Phenol groups

Many phenol groups have been explored in wet adhesion, including monophenol-based tyrosine [37,38], the maritime creatures-inspired DOPA chemistry [39,40], and polyphenols like tannic acid and pyrogallol [[41], [42], [43]]. Synthetic or natural compounds like polyethylene glycol (PEG), gelatin, chitosan, hyaluronic acid, dextran and alginate can be purposed for modification with phenol groups [32]. The presence of phenol groups ensures the formation of covalent or non-covalent crosslinking with tissue proteins under wet conditions. At the same time, they can self-crosslink or react with other amino-contained compounds to form cohesion. To creat cohesion, irreversible crosslinking strategies, like NaIO_4_, AgNO_3_, KMnO_4_, HRP/H_2_O_2_, fibrinogen, hematin, MgO and tyrosinase, were used to oxidize phenol groups to form chemical crosslinking with tissues and themselves (Fig. 2A) [[44], [45], [46], [47], [48]]. Reversible crosslinking strategies, like Fe^3+^ crosslinking and borate crosslinking, have also been used [48,49]. The advantage of using reversible bonds to fabricate the cohesion is that the bioadhesives can easily have properties owned by cleavable bonds, like responsive and self-healing properties. For instance, with DOPA modified dendritic polydextran (PDA) polymer, Zhang et al. have reported an adhesive based on a tunable bone for sternal closure. They found that low Fe^3+^ to DOPA ratios offered strong but reversible interactions [48]. Shan et al. used borate to form reversible covalent bonds with catechol groups to fabricate a pH, glucose, and dopamine triple-responsive and self-healable bioadhesive [50].

An important function that this strategy can apply to bioadhesives is the antibacterial property. By redox reaction, polymers containing phenol groups can transform silver nitrate into silver nanoparticles, which have been widely used as effective antibacterial agents. So, Guo et al. fabricated injectable citrate-based mussel-inspired bioadhesives [46]. Silver nitrate was used to oxidize the DOPA-modified citrate to form the cohesion and simultaneously the silver nanoparticles. To further improve the anti-microbial property, they incorporated 10-undecylenic acid into the polymer chain. The resulting bioadhesives showed effective anti-microbial properties against *Escherichia coli*, *Staphylococcus aureus* and *Candida albicans*. Later, they used silver nitrate to oxidize tannic acid-modified gelatin and fabricated silver-releasing anti-microbial bioadhesives [51]. Another mechanism for killing bacteria with phenol groups is photo-thermo-induced bacterial death. Photothermal therapy has been used for killing multi-drug resistant bacteria because enzymes of bacteria will be denatured, and proteins and lipids on the bacteria cell membranes will be destroyed at high temperatures [52]. Catechol groups have good photothermal capacity after complexing with Fe^3+^. Inspired by this, Zhao et al. fabricated physical double-network adhesives with antibacterial properties [27]. It was shown that catechol-Fe^3+^ coordination crosslinked hydrogels possessed excellent photothermal capacity and antibacterial activity against gram-positive and negative bacteria.

Although phenol groups have been among the most widely explored adhesion mechanisms, there are still many concerns relating to their commercialization. For example, the prohibitive costs and the potential neurological effects of DOPA limit their commercialization in wound closure [51,53]. Another problem is that oxidation of phenol groups usually generates dark/brown color. Although it was observed in plenty of papers that the dark brown color no longer exists after the wound healing, no data showed how exactly these oxidized phenol compounds would be metabolized inner the body.

#### 2.1.2 NHS-ester

In organic chemistry, an active ester is an ester functional group that is highly susceptible to nucleophilic attack. NHS-ester is the most important activated ester used in many different bioconjugation techniques, such as protein labeling and peptide synthesis [54]. Since NHS-ester, which is very bio-safe, has high selectivity towards primary amines and thiol groups under mild conditions, it has been used to fabricate bioadhesives by forming covalent bonding efficiently with amino and thiol groups from tissues (Fig. 2B) [55]. Besides, it is also easy to find biocompatible and functional compounds with amino and thiol groups for cohesion, like gelatin, peptide dendrimer, lysozyme, collagen, bone marrow, chitosan, polyallylamine and amino-terminated PEG [25,[56], [57], [58], [59], [60], [61], [62]]. As a result, NHS-ester has also been used for cohesion.

Usually, the reaction of NHS-ester generated amido bonds, which are very stable, resulting in good cohesion strength. Most of the NHS-ester-based adhesives have been used as sealants [25,26,28,60,[62], [63], [64], [65]]. The dural repair sealant DuraSeal® and vascular sealant Coseal® are active ester-based adhesives with polylysine and thiol terminated PEG for the cohesion. Another attractive property for active ester-based cohesion is the controllably dissolvable property through the formation of either thioester or succinyl ester. Ghobril et al. used the reaction between the active ester and thiol groups to fabricate a thioester-based sealant [28]. Using the thioester exchange reaction, the bioadhesives showed controllably dissolvable property when immersed in ʟ-cysteine methyl ester solution. In our previous work, it was found that the reaction between succinimidyl succinate-based active ester and amino groups could be used to fabricate succinyl ester-based sealants, which showed controllable dissolution and good biocompatibility [25]. It was demonstrated that the sealants showed good hemostatic property without causing sides effects in a rabbit lung injury model.

For NHS-ester-based bioadhesives, the NHS-ester should already be in the compounds before application. For example, PEG NHS-esters are components that are ready-to-use for DuraSeal® and Coseal®. However, because of the easy hydrolysis of NHS-ester, it often needs careful storage, limiting its shelf life and increasing the cost. So there are strategies to use carboxyl groups (COOH)-containing compounds to form active ester *in situ* when applying the bioadhesives by adding catalysts 1-Ethyl-3-(3-dimethylaminopropyl)carbodiimide (EDC) [25,66,67]. Pinkas et al. used natural polymers gelatin and alginate to fabricate bioadhesives. To form the cohesion, they mixed EDC with the polymers solution prior to use. EDC will catalyse the reaction between COOH from alginate/gelatin and NH_2_ from gelatin for cohesion. Although this method avoids the storage of NHS-ester, it caused safety concerns because of the cytotoxicity of residual EDC.

#### 2.1.3 Aldehyde

Aldehyde groups can quickly form Schiff-base linkages with amino groups from tissues and other compounds [68]. The oxidation of polysaccharides using periodate is an easy way to generate aldehyde groups. So some of the polysaccharides such as chitosan, dextran, alginate, hyaluronic acid and chondroitin sulfate are oxidized to generate the aldehyde-based bioadhesives [29,[68], [69], [70], [71], [72], [73], [74], [75], [76], [77], [78], [79], [80]], followed by crosslinking with another amino-containing crosslinker, like chitosan, gelatin, ployethylenimine (PEI), polypeptide, amino terminated PEG and polyvinyl alcohol co-vinylamine (PVA-A) for cohesion (Fig. 2C) [29,[72], [73], [74],^76^,^77^]. Giano et al. used NaIO_4_ to oxidize dextran to generate aldehyde groups and then crosslinked the oxidized polymer with PEI [72]. They found that the adhesive showed an adhesion strength of ∼2.8 kPa and significantly improved animals' survival rate in a cecal ligation and puncture model. The commercially available Bioglue®, fabricated by albumin and glutaraldehyde, is another example of bioadhesives using this strategy, indicated for vascular sealing. In fact, Schiff base linkages formed by aldehyde and amino are among the most widely used methods to prepare smart biocompatible hydrogels with self-healing properties. Moreover, Schiff bases cleave at acid pH so that pH-responsive bioadhesives can be fabricated with this strategy [[81], [82], [83]]. Bioadhesives, of which cohesion is created by aldehyde groups, theoretically have self-healing and pH-responsive properties. The self-healing cohesion can be used to fabricate bioadhesives with self-healing adhesive strength, which might be helpful in wound dehiscence [61]. The pH responsiveness can endow the bioadhesives with controllably removable properties. But this strategy was tried in only a few bioadhesives, which might have resulted from the slow rate of self-healing or pH responsiveness.

#### 2.1.4 Cyanoacrylate

In 1949, cyanoacrylates were first synthesized by a German chemist and used in wound closure ten years later [84]. They demonstrate strong and rapid-acting adhesive properties in seconds, with different formulations approved by the FDA for biomedical applications. In the presence of hydroxide ions, cyanoacrylates undergo exothermic polymerization for cohesion (Fig. 2D). At the same time, adhesion forms through the covalent bonds between cyanoacrylates functional groups in tissue proteins [35,85,86]. However, exothermic reaction, concerns about the toxicity of degradation products, and lack of required flexibility for soft tissue adhesion put limitations on their applications, especially for internal ones [2,30,35,87]. The efforts in developing novel cyanoacrylate-based bioadhesives have focused on solving those problems. Basu et al. used PEG400 biscyanoacrylate as a crosslinker to copolymerize with 2-octyl cyanoacrylate. It was proved that the resulting bioadhesives showed increased plasticity, mechanical strength and resilience [88]. By combining DOPA, Lim et al. got allyl 2-cyanoacrylate-based bioadhesives with toxicity 1.5 times lower than that of pure allyl 2-cyanoacrylate when tested against L929 cells by using the direct contact methods [89]. It is also worth noting that although cyanoacrylate-based bioadhesives produce potential side effects, among the commercialized bioadhesives, including fibrin, PEG, gelatin and albumin-based ones, they have the strongest adhesive strength, which is indicated for wound closure.

#### 2.1.5 Click chemistry

Click chemistry is a class of reactions that has been extensively used to fabricate hydrogels rapidly [90,91]. Click reactions have high thermodynamic driving forces (>20 kcal/mol), which enable these reactions to proceed rapidly to completion with high selectivity [92]. Considering the high reactivity of the adhesive functional groups, click crosslinking with high selectivity is a good choice for cohesion fabrication without influencing the other reactions in the bioadhesive system. Three types of click chemistry are commonly used in bioadhesives, including DA, AAC, and Thiol-ene reactions (Fig. 2E). Pramudya et al. developed glucose-based bioadhesives. In their system, catechol groups contributed to the adhesion while AAC was used to enhance cohesion [93]. The adhesives had strong adhesion on biological surfaces with structural similarity to that of natural carbohydrate, water compatibility and control lability of adhesion strength. In Pramudya's work, click reaction is the main reason for cohesion. Because of high selectivity, it can also be used with other crosslinking to form double crosslinked cohesion. Guo et al. fabricated citrate-based mussel-inspired bioadhesives [18]. NaIO_4_ has been used to oxidize the catechol groups to form cohesion in their design. To further increase the cohesion, copper-catalyzed AAC was used to improve the bioadhesives' cohesive strength, resulting in improved adhesion strength. To avoid the mutual inhibition effect between NaIO_4_ and click catalyst, a zwitterionic organic chemical buffering agent, 4-(2-hydroxyethyl)-1-piperazineethanesulfonic acid (HEPES), which is widely used in cell culture as a buffering agent, was used. Later they found that the dual-crosslinked iC-X-PI bioadhesives expressed significantly enhanced cohesive and adhesion strength. However, aware that copper catalysts are often harmful to cells, Li et al. chose the copper-free click chemistry pair tetrazine/*trans*-cyclooctene (TZ/TCO) to modify chitosan [94]. TZ/TCO possesses high chemoselectivity and ultrafast kinetics and is very stable in an aqueous solution under physiological conditions. Together with the Schiff bases moieties introduced by aldehyde modified PEG for cohesion, TZ/TCO increased the cohesion strength, leading to an adhesion strength which is 2.3-fold that of fibrin glue. DA reaction is another kind of copper-free click chemistry that is rapid, efficient, versatile, selective and widely used in tissue engineering [95,96]. Yu et al. modified hyaluronic acid (HA) with adipic dihydrazide, furylamine and aldehyde groups to get HA-furan-ADH HA-furan-CHO. After mixing these two components, a double network is formed, including Schiff base moieties and click chemistry crosslinking, with even more outstanding adhesion and cohesion strength [79].

#### 2.1.6 Photo-crosslinking

A light stimulus is considered an ideal external control to manipulate hydrogels' properties, which brings advantages such as ease of control by switching on/off, requiring milder conditions for crosslinking, and tunable biochemical and biophysical properties [[97], [98], [99]]. An advantage of photo-crosslinking in bioadhesives is that it will not influence the reaction of most functional groups for adhesion (FGA) (Fig. 2F). DOPA is one of the most common strategies explored to be used with photo-crosslinking. Xuan et al. used methacryloyl and DOPA modified gelatin to fabricate a nanosheet adhesive to treat acute trauma. Crosslinking of methacryloyl groups-modified gelatin is responsible for cohesion, while DOPA contributes to adhesion [100]. The adhesive showed effective adhesion to irregular tissues in a wet condition, and the adhesion strength can easily be tuned by varying the amount of DOPA functional groups. Li et al. fabricated adhesives based on acryloyl and DOPA modified PEG and methacrylate modified dextran by photo-crosslinking [101]. Later, this way optimized the system by varying the DOPA content and achieved a maximum bursting pressure of 620 mmHg.

Because of the ability to form a double network, photo-crosslinking has been used to fabricate bioadhesives with strong cohesion strength. The double-network strategy uses the mechanism in which the energy is dissipated by breaking the sacrificial bonds and is widely used in the fabrication of hydrogels with formidable mechanical strength [102]. By using this concept in the cohesion of bioadhesives, Li et al. designed a series of adhesives. The cohesion was formed by a double-network of calcium ion-crosslinked alginate photocrosslinked polyacrylamide network [6]. Under stress, the crosslinked alginate can be broken first to dissipate energy. Adhesion was separately designed mainly by the reaction of carboxyl groups and amino groups under EDC/NHS catalysis. It was reported that the adhesion occurred within minutes, and the strength was greater than that of the commercialized Cyanoacrylate, Coseal® and a nanoparticle-based adhesive. Yuk et al. fabricated dry double-sided tape for the adhesion of wet tissues and devices [8]. They used photocrosslinking of acrylic acid and acrylic acid active ester to form the first network of the adhesive and biopolymers (for example, gelatin or chitosan) to form the second network for energy dissipation. For adhesion, the active ester can form covalent bonds with the tissues. Their wet adhesion mechanism was realized by removing the interfacial water from the tissue surfaces by swelling the adhesive. The final adhesives showed an adhesion strength more significant than that of the commercialized Histoacryl®, Dermabond®, BioGlue®, Coseal®, DuraSeal®, Tisseel® and Tegaderm® hydrocolloid. Li and Yuk's two bioadhesives, with double-network strategies, both use active ester as the adhesion mechanism. In contrast, few active ester-based bioadhesives were previously reported to have a comparable adhesion performance. One cause of this difference is the strong cohesion formed by the double-network strategy.

Not only because they are good at making adhesives patches, a further benefit of the double-network strategy is that it can make photo-crosslinking-based bioadhesives injectable. Importantly, avoiding the precursor solution flow to other sites before the light-induced polymerization is a problem for photo-crosslinked bioadhesives. To make those bioadhesives injectable, the double-network-based sequentially crosslinking strategy was used. A first network formed fast to support the whole structure and then photo-crosslinking formed as the second network to further enhance the bulk strength [74,103]. Jeon et al. have used 2-aminoethyl methacrylate to modify oxidized alginate which resulted in a methacrylate and aldehyde-modified compound (OMA) [74]. First, amino-terminated PEG was used to crosslink OMA to form the first network by Schiff bases and later photo-crosslinking to form the second network. The resulting system had dual crosslinking of Schiff bases and photo-crosslinking, leading to good adhesion strength and cytocompatibility. Using precursors with high viscosity also increases the injectability of photo-crosslinking-based bioadhesives. Hong et al. combined methacrylate modified gelatin and *N*-(2-aminoethyl)−4-(4-(hydroxymethyl)−2-methoxy-5-nitrosophenoxy) (NB) modified hyaluronic acid [103]. The whole system can be directly injected. With photo-crosslinking, the NB modified hyaluronic acid forms covalent Schiff bases bonds with gelatin's amino groups, and simultaneously, there was a photo-crosslinking of methacrylated gelatin. The developed bioadhesive withstood blood pressure up to 290 mmHg and efficiently stopped high pressure bleeding from both pig carotid arteries and hearts bleeding models.

A concern with photo-crosslinking is that most of the photo-crosslinking-based bioadhesives are UV initiated, with the potential of causing photochemical cytotoxicity or DNA damage [104]. A good way to solve this problem is to use initiators that can be initiated with visible light. Sani et al. used methacrylic anhydride-modified gelatin to develop bioadhesives for sutureless repair of corneal injuries [105]. To avoid the side effects of UV crosslinking, they used photoinitiators, Eosin Y, triethanolamine (TEA) and *N*-vinylcaprolactam (VC), which can be crosslinked after short exposure to visible light (450 and 550 nm). It was found in a rabbit stromal defect model that the bioadhesives effectively seal corneal defects and induce stromal regeneration.

### 2.2 Non-covalent strategies

#### 2.2.1 Phase transition

Phase transition is a physical process of transition between the basic states of matter from a solid, liquid or gas state to a different state. It has been rigorously studied as a rapid *in situ* gelling systems, especially for those systems showing sol-gel transition behaviours at a temperature near 37 °C [106,107]. Using those polymers with a critical solution temperature at physiological temperature, injectable bioadhesives can be easily fabricated (Fig. 2G). Poly(ethyleneoxide)-poly(propylene oxide)-poly(ethylene oxide) (PEO-PPO-PEO, Pluronic) is one of the commonly used temperature-responsive polymers. Lee et al. used DOPA modified hyaluronic acid and thiol terminated Pluronic copolymer to fabricate injectable bioadhesives [21]. The adhesive was formed by the Michael-type addition between DOPA and thiol groups, which further showed high cohesion integrity at 37 °C because of the phase transition of Pluronic polymer. DOPA modified chitosan has also been used to combine with Pluronic to fabricate bioadhesives with injectability and superior hemostatic properties [22]. Except for Pluronic, poly(*N*-isopropyl acrylamide) (PNIPAAm) was also used to enhance cohesion via phase transition, which can form a compact hydrogel at physiological temperature [78].

#### 2.2.2 Hydrophobic interactions

Hydrophobic association is one of the strategies for improving hydrogels' toughness, which has also been used in strengthening the cohesion mechanism of bioadhesives (Fig. 2H) [108]. Gao et al. introduced hydrophobic segments into DOPA-based bioadhesives, enhancing the network due to effective energy dissipation [109]. In their design, FeCl_3_ crosslinked DOPA (complexation) can work synergistically with the hydrophobic interaction, resulting in bioadhesives with a mechanical strength of 30 kPa and high extensibility of 2000%. Because of the reversible property of catechol-Fe^3+^ complexes and hydrophobic association, the system also exhibited self-healing behaviour. Besides, catechol-Fe^3+^ complexation is pH-dependent and hydrophobic association is temperature-sensitive, so resulting bioadhesives showed both pH and thermo responsiveness. Another interesting part of hydrophobic interactions is that they can turn the bioadhesives' swelling since the hydrophobic part in the system can inhibit the water absorption.

#### 2.2.3 Doping

A phenomenon often observed in nature is that tissues are supported by fibres to provide mechanical reinforcement, like articular cartilage, mammalian cells and even some living organs [110]. Inspired by this, doping has been used increase the cohesiveness of the adhesives (Fig. 2I). Pinkas et al. incorporated cellulose fibers into the network to reinforce the polymeric matrix [111]. They found that together with gelatin and alignate, the fibre-reinforced adhesive showed superior mechanical and physical properties, and thus has great potential in acting as a surgical sealant because of the improvement in cohesive strength. Nanoparticles have also been used to fabricate mechanically strong hydrogels, which can be used in strengthening the cohesion of bioadhesives [112,113]. Liu et al. combined Laponite nanoparticles with DOPA chemistry, which is degradable and can promote type I collagen synthesis [114]. They found that adding laponite significantly reduced the curing time and enhanced the bulk mechanical and later the adhesion strength. Furthermore, those nanoparticles even improved the cell affinity of the bioadhesives. Because laponite solution can lead to the autooxidation of dopamine and the subsequent covalent crosslinking of catechol residues, it was also used to induce the oxidation of DOPA oxidation and act as the cohesion enhancing strategy, resulting in a bursting pressure of 320 mmHg [115]. Unlike laponite, PLGA is biodegradable. Pandey et al. incorporated PLGA nanoparticles into the DOPA-modified alginate polymer and the adhesives formed by oxidation with NaIO_4_ [116]. The PLGA was also modified with NHS to further increase the adhesion strength, which was more than two-fold that of the control without PLGA nanoparticles [116]. As well as their contribution to the cohesion strength, nanoparticles can also increase the adhesion thanks to their ability to adsorb onto polymer chains and form bridges between two connecting structures [117]. As a result, doping of nanoparticles can be a method for enhancing both the adhesion and cohesion. However, considering that doping alone cannot support the bulk configuration of the bioadhesives, it is always used as an adjunct to enhance the cohesion strength.

#### 2.2.4 Hydrogen bonds

Hydrogen bonds are electrostatic attractions between two polar groups when a hydrogen atom covalently bound to a highly electronegative atom experiences the electrostatic field of another highly electronegative atom, which has also been used to form the cohesion of the bioadhesives (Fig. 2J) [118]. Because of the reversible properties of hydrogen bonds, the resulting bioadhesives usually have self-healing properties. One typical example is the use of degradable tannic acid, a weakly acidic polyphenolic compound containing digallic acid groups conjugated to a central glucose core via ester linkages [41,119]. It forms a complex or crosslinks with macromolecules through multiple interactions, including hydrogen and ionic bonding, and hydrophobic interactions [120]. By using tannic acid and PEG, Sun et al. fabricated PEG active ester and tannic-based adhesives [61]. The hydrogen bonds were responsible for the cohesion, while active ester was responsible for the adhesion. Because the hydrogen bonds have self-healing properties, they found that their adhesives had self-healing adhesion strength. However, it is worth noting that the dissociation energy of a single H-bond is low. To make the cohesion strong enough, multiple H-bonds in a single domain are usually needed [41,121]. This is also why tannic acid with a multi-hydrogen bonding site in a single molecule is a popular crosslinking agent in this strategy [42,122].

#### 2.2.5 Self-assembly

As is defined in *Nature Portfolio*, self-assembly is the process by which an organized structure spontaneously forms from individual components as a result of specific, local interactions among the components. Self-assembly is also an essential process in natural underwater adhesive systems [123,124]. Those adhesives are secreted from the secretory organ to the targeted place, then undergo self-assembly in a non-covalent fashion for the bulk functions and finally cure to a suitable toughness. Inspired by this, bioadhesives have also been developed by using self-assembly (Fig. 2K). By combining two independent natural adhesion systems, which were mussel foot proteins (Mfps) adhesives and CsgA-based adhesives, Zhong et al. designed a new generation of bio-inspired adhesives [125]. These hybrid molecular materials hierarchically self-assembled into higher-order structures, in which, according to molecular dynamics simulations, disordered adhesive Mfp domains were exposed on the exterior of amyloid cores formed by CsgA. In their systems, amyloid fibre structures enabled large surface areas for contact, with multiple disordered Mfp domains on fibre surfaces interacting to achieve enhanced ultra-strong underwater adhesion. As a result, the bioadhesive had an underwater adhesion energy approaching 20.9 mJ m^−2^, which is 1.5 times greater than the maximum of reported bio-inspired and bio-derived protein-based underwater adhesives.

## 3 Relationships between adhesion and cohesion

**Fig. 3:**
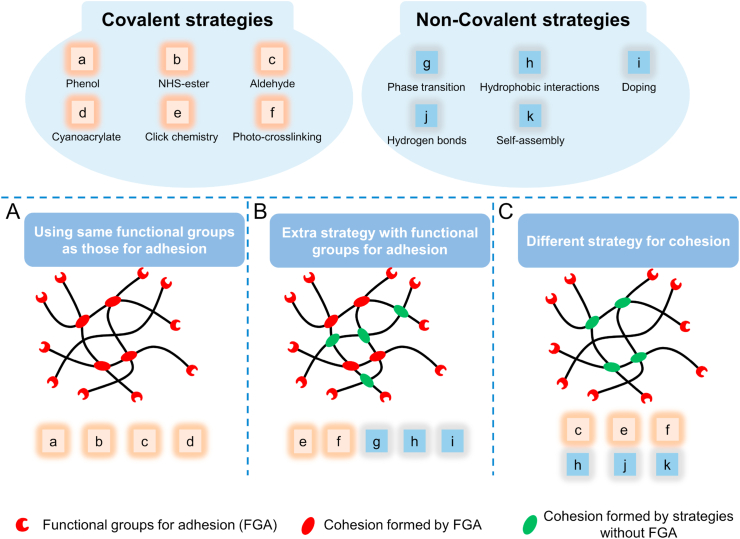
**The relationships between adhesion and cohesion.** The relationships between adhesion and cohesion can be grouped as: A, Using the same functional groups for cohesion and adhesion including (a) phenol groups, (b) NHS-ester, (c) aldehyde groups and (d) cyanoacrylate-derivatives-based strategies; B, Using extra strategy with functional groups for adhesion including (e) click chemistry, (f) photo-crosslinking, (g) phase transition (h) hydrophobic interactions and (i) doping-based strategies; C, Using a different strategy for cohesion including (c) aldehyde groups, (e) click chemistry, (f) Photo-crosslinking, (h) hydrophobic interactions, (j) hydrogen bonds and (k) self-assembly-based strategies.

When designing a bioadhesive for certain applications, choosing the adhesion mechanism is the first step for most researchers. Then, a cohesion strategy will be applied accordingly to finally form the bioadhesive. So, understanding the relationship between adhesion and cohesion will help in the design of bioadhesives, finally facilitating the translation. According to the authors' understanding of bioadhesives, the relationship between cohesion and adhesion can be grouped into three categories: 1) Using the same functional groups for cohesion as those for adhesion (Fig. 3A); 2) using extra functional groups for cohesion in addition to groups to adhesion (Fig. 3B); 3) Using different strategies for cohesion (Fig. 3C). The commonly used cohesion strategies (covalent and non-covalent ones) have also been grouped according to the main mechanisms of different bioadhesive systems.

**Fig. 4:**
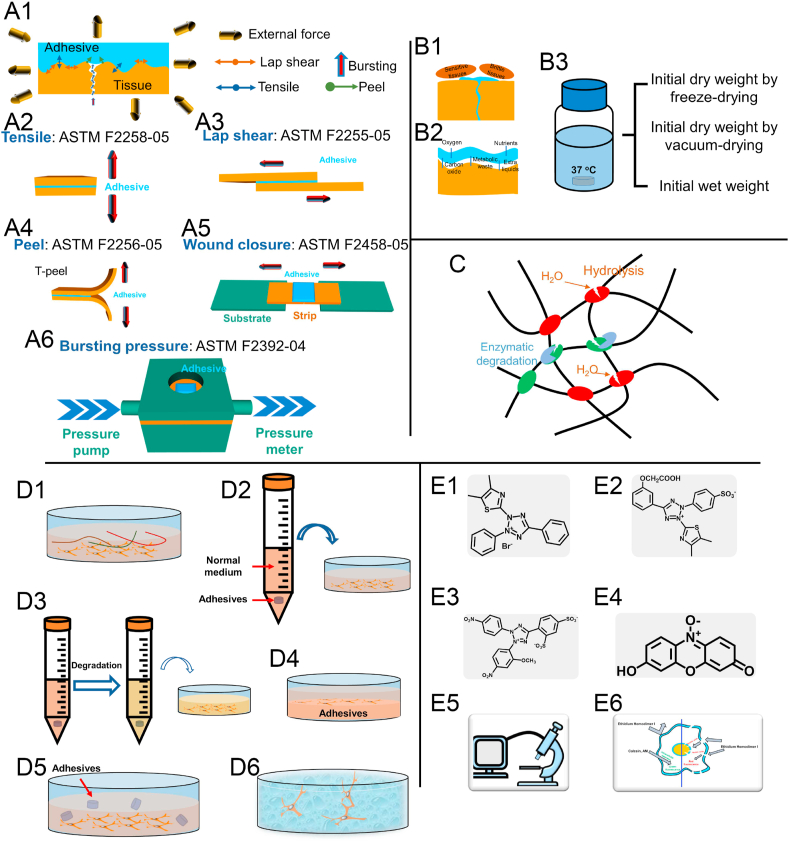
***In vitro/ex vivo* models developed for the analysis of bioadhesive systems.** A1, Schematic showing that after adhering bioadhesives to the tissues, the external forces might come from different directions and the adhesion interfaces will undergo different breakage mechanisms. A2 to A6, Schematic showing five commonly used adhesion performance evaluation methods, including (A2) tensile (ASTM F2258-05), (A3) lap shear (ASTM F2255-05), (A4) peel (ASTM F2256-05), (A5) wound closure (ASTM F2458-05), and (A6) bursting pressure (ASTM F2393-04) tests. B1, Schematic showing swelling leads to extra pressure to brittle or sensitive tissues. B2, Schematic showing swelling is good for gas and nutrients transfer and exchange. B3 Three ways to calculate the initial weight in swelling ratio tests. C, Degradation by hydrolysis and enzymatic degradation. D, Schematic showing the ways to evaluate the cytotoxicity of the bioadhesives, including (D1) leachable contents, (D2) prepolymer contents, (D3) degradation products, (D4) seeding cells on the surface of the bioadhesives, (D5) coincubation as well as (D6) encapsulation of cells into the bioadhesives. E, Six commonly used assays to assess the outcome of the cytotoxicity tests, including, (E1) 3-(4,5-Dimethylthiazol-2-yl)-2,5-Diphenyltetrazolium Bromide (MTT) assay, (E2) 3-(4,5-Dimethylthiazol-2-yl)-5-(3-carboxymethoxyphenyl)-2-(4-sulfophenyl)-2H-tetrazolium (MTS) assay, (E3) Cell Counting Kit-8 (CCK-8) assay, (E4) AlamarBlue®/PrestoBlue, (E5) photomicroscopy as well as (E6) Live & dead assay.

### 3.1 Using same functional groups

Most of the bioadhesives' adhesion relies on the interactions between FGA and tissues. Those FGA are reactive, so they also self-crosslink or crosslink with certain crosslinkers to form the bulk network of the bioadhesives (Fig. 3A). Most of the bioadhesives using phenol groups, NHS-ester, aldehyde and cyanoacrylate for cohesion can be grouped in this category. For example, phenol groups are sticky because they interact with tissues through covalent or non-covalent crosslinking. Meanwhile, irreversible and reversible strategies, including oxidation and complexation, have also been applied to induce phenol groups' self-crosslinking for cohesion. Active ester reacts with amine and thiol groups from tissue proteins for adhesion. Since there are plenty of amino or thiol groups-functionalized compounds, the combination of active ester and those groups contributes to the cohesion of those bioadhesives.

Since modification of the compounds with different functional groups is complicated, involving different reaction conditions and the possibility of interference of different functional groups, the advantage of using FGA for cohesion is that the focus can be entirely on adhesion without the need to design another crosslinking mechanism for adhesion, thus contributing to the ease of fabrication. Most of the commercially available bioadhesives including fibrin-based (Tisseel®), PEG-based (Coseal®, DuraSeal®), cyanoacrylate-based (Histoacryl®, Dermabond®) and albumin-based (BioGlue®, Progel™) belong to this group. Besides, an important feature for using the same functional groups for adhesion and cohesion is that most of them are *in situ* injectable bioadhesives, which can be described as bioadhesives which the cohesion and adhesion formed at the same time. So, it is easy to fabricate injectable bioadhesives with this strategy. However, because the number of functional groups for a given formulation is usually constant, there will be a competition between adhesion and cohesion [2]. Take the DOPA chemistry as an example; if more DOPA groups are used for cohesion, fewer are available to crosslink with the tissues for adhesion. So, it is always vital to find a balance between adhesion and cohesion by varying the ratio of crosslinkers and polymer composition [47,126]. Moreover, there is a concern that if some of those bioadhesives do not contact the tissues shortly after the injection, most of the bioadhesives using this strategy will lose their adhesion strength because the FGA will all be consumed by forming the cohesion.

### 3.2 Using combinational approaches together with FGA

Cohesive strength is crucial for the final adhesion strength. Take DOPA chemistry for example. DOPA-based adhesives suffer from insufficient cohesive strength under wet conditions, which quickly leads to adhesion failure [18,19]. Cohesion reinforcement has been considered when bioadhesives are explored further in load-bearing tissues or elastic and soft tissues, where they must withstand pressure and stress [17,111]. To reinforce the cohesion, some bioadhesives use combinational approaches together with FGA for cohesion (Fig. 3B), which falls into this category [17,127,128]. In this case, the extra force will have little influence on the adhesion mechanism but strengthen the cohesion by offering extra interactions. Generally, the additional force includes covalent and non-covalent strategies (Fig. 3B).

*Covalent strategies*. Click chemistry is a widely used strategy here because of its high selectivity. It has been used to combine with DOPA, Schiff bases to fabricate the bioadhesives, where it enhanced the cohesion without interfacing FGA. Photo-crosslinking is another covalent strategy acting as an additional approach. Generally, photo-crosslinking is difficult to fabricate injectable bioadhesives because the precursor solutions easily flow away before the crosslinking happens. However, in this strategy, the cohesion formed by the FGA easily supports the network to stay on-site and there is enough time left for photo-crosslinking to enhance the cohesion [74,103]. In this way, photo-crosslinking in this strategy is also used to fabricate injectable bioadhesives.

*Non-covalent strategies*. Non-covalent enhancement using strategies with less reactivity, thus influencing little on FGA. Sol-gel transition, hydrophobic interactions and even nature-inspired doping have been combined with FGA for cohesion. In Lee's work, phase translation of PEO-PPO-PEO enhanced the cohesion formed by Michael-type addition between DOPA and thiol groups [21]. In Gao's work, hydrophobic interaction was introduced to enhance FeCl_3_-crosslinked DOPA by acting as a way for effective energy dissipation [109]. Also, doping of fibre structure and nanostructure has been explored in strengthening the original cohesion formed by FGA [111,114].

Using additional force in the bioadhesive network is a way to decrease competition's side effects caused by FAG for cohesion and adhesion. The cohesion can be significantly enhanced by the second force, leading to an increased final performance. Non-covalent strategies can be quickly introduced by modification or doping, some of which can even endow the system with more functions, including temperature-responsive and self-healing properties. Covalent strategies usually result in stronger bonds by further increasing the crosslinking strength. However, the design should avoid the toxicity caused by the different functional groups or dopants and the interference of those with FGA. Except for click chemistry and photo-crosslinking, few other covalent strategies have been explored together with FGA.

### 3.3 Using different strategy for cohesion

In addition to FGA, there are many other strategies to fabricate hydrogel systems with proper bulk mechanical strength. By using these strategies, the competition between adhesion and cohesion can be directly avoided (Fig. 3C) [129]. Properties of cohesion can be purposely designed by selecting the relevant strategies.

*Covalent strategies.* Photo-crosslinking is the most widely used strategy here. It can easily be combined with DOPA chemistry because these do not interfere with each other. In Xuan's work, methacryloyl and DOPA modified gelatin were used to fabricate a nanosheet adhesive. Crosslinking of methacryloyl groups is responsible for cohesion, while DOPA only contributes to adhesion [100]. Aldehyde-based Schiff base reaction has also been combined with DOPA chemistry to fabricate bioadhesives, in which Schiff base is mainly responsible for the cohesion and DOPA for the adhesion [23,130]. However, since DOPA will induce more excellent bonding with tissues after oxidation and the Schiff base bonds are not strong, the combination of Schiff base bonds and DOPA will induce less adhesion strength than directly oxidizing the DOPA groups [23]. Because of high selectivity, Click chemistry was also used alone for cohesion. Combined with DOPA chemistry, Pramudya et al. used Azide-Alkyne cycloaddition to fabricate the cohesion for the DOPA-based bioadhesives [93].

Non-covalent strategies. Non-covalent strategies, like hydrophobic interaction [24], self-assembly [125,131,132], and phase translation [133,134], have also been used for the cohesion of bioadhesives. Cui et al. fabricated a hyperbranched polymer with a hydrophobic backbone and hydrophilic adhesive catechol side [24]. Upon contact with water, the hydrophobic chains self-aggregated to form cohesion and displace the water. The catechol groups were exposed to substrates for adhesion. Brennan et al. used elastin-like polypeptide bioadhesive produced by *Escherichia coli* [133]. The cohesion resulted from the material phase transition, which could be tuned to coacervate in physiological conditions. Although non-covalent strategies' cohesion strength is less than that of covalent strategies, an appealing aspect of these non-covalent strategies is that they can readily offer injectability to the bioadhesives.

By separating the cohesion and adhesion mechanism, the design of bioadhesives becomes an ‘assembly project’, and the competition between adhesion and cohesion is directly avoided. Take the above-mentioned double-network adhesives as an example [6,8]. NHS-ester is the group for adhesion. Before their reports, there was almost no work showing that NHS-ester would induce so great an adhesive strength. As discussed above in the photo-crosslinking section, one reason is that the strong cohesion offered by the double-network strategy. Another possibility might be that all NHS-ester can be used for adhesion in their work. As a result, depending on the different applications, it is only necessary to choose the right cohesion and adhesion strategies, like hydrogen bonds for self-healing adhesives, phase transition for injectability, and photo-crosslinking strategy for a robust adhesive patch [93,131].

## 4 Perspective and conclusion

As with indispensable everyday adhesives, bioadhesives also have great potential in biomedical applications. Over the past decades, great attention from scientists has been placed on different kinds of bioadhesives. The applications focused on wound closure, sealing leakage and immobilization, aiming to decrease the complications for patients and increase the beneficial outcome of tissue repair/regeneration. Although many bioadhesives in papers showed promise, few reached the market and there are only traditional cyanoacrylate, PEG, fibrin, gelatin and albumin-based bioadhesives for routine applications in the market.

One reason might be that too much attention was paid to developing a new adhesion mechanism, while the cohesion mechanism was ignored. Cohesion is especially important for adhesive strength. Since breakage of the adhesion is an energy dissipation process, adding extra force into the original network is a way to improve cohesion. Those forces include covalent strategies like click chemistry and photo-crosslinking, which do not interfere with most of the FGA. Non-covalent forces including doping, phase-transition and hydrophobic interactions have been explored to enhance the network. Also, separating the adhesion from cohesion is a good strategy, among which double-network induced powerful adhesion strength.

Cohesion strategies will also contribute to the therapeutic outcome. A good example is the antioxidant property of phenol groups. Overproduction of reactive oxygen species (ROS) in wounded areas would cause oxidative stress to surrounding tissues and hinder tissue healing. Phenol groups were reported to act as a ROS scavenger to protect cells from oxidative stress [135]. Inspired by this, Liang et al. used gelatin-grafted-dopamine and polydopamine-coated carbon nanotubes to engineer a hydrogel adhesive [136]. They found that bioadhesives expressed a good antioxidant property by using DPPH assay and significantly accelerated wound healing in a full-thickness mouse skin defect model. Embryonic wound healing-inspired mechanically active adhesive is another excellent example, in which the formation of actin cables applies force to contract the wound edges together [137]. By using poly(*N*-isopropyl acrylamide) (PNIPAm) and photo-crosslinking, Blacklow et al. added thermoresponsiveness to their bioadhesive patch's cohesion design [138]. After being applied to the wound, the patch would draw the wound together because of the shrinkage of PNIPAm at a temperature of more than 32 °C. The efficacy in accelerating skin wound healing was demonstrated using the full-thickness excision wound.

Cohesion strategies directly determine how the bioadhesives will be used. For bioadhesives, strong adhesion strength is an important but not the essential character for translation. Take bioadhesive-based wound dressing for example. An adequate adhesion strength for stable immobilization is enough since strong adhesion may cause a problem for later health care, such as in the changing of wound dressing or re-exposure of the wound [139]. And in many cases, ease of use directly decides if the patients or the doctors are willing to use the products, which is decided by cohesion. For bioadhesives to cover irregular wounds, it would be preferable to be *in situ* injectable to cover every corner of the wound. For bioadhesives for cell loading, the bulk strength will significantly influence the cells' viability inside, which the cohesion mechanism can tune. Smart removable and self-healing properties for ease of use can also be realized through cohesion design, increasing their adaptability [29,139,140].

Finally, one bioadhesive cannot satisfy all the applications. Every bioadhesive system should be developed with a specific target, and the target directly decides the properties needed. Cyanoacrylates perform well in wound closure but are very weak in sealing, and whereas albumin-based Bioglue® works oppositely. The tensile or peel strength should be the larger, the better for wound closure, but high cohesion seems to be more critical for sealant. For applications in an environment with a large amount of liquids like inner blood vessels, a preformed patch is more appropriate than *in situ* forming bioadhesives. As a result, choosing the proper characterization methods according to the applications is also essential. Take the adhesion performance evaluation for example. There are five different testing guidelines designed by American Society for Testing and Materials (ASTM), including tensile (ASTM F2258-05), lap shear (ASTM F2255-05), peel (T-peel) (ASTM F2256-05), wound closure (ASTM F2458-05) and bursting pressure (ASTM F2392-04) tests (Fig. 4A1 to A6). The wound closure strength is critical for the tape-like bioadhesives, tensile, peel, or lap-shear strength is vital for glue-like bioadhesives and bursting pressure is critical for sealants.

In addition to adhesion performance evaluation, how to test swelling ratio, degradation and cytotoxicity tests should also be decided according to the targeted application and bioadhesives' formulations (Fig. 4B–E). The swelling ratio is defined as the fractional increase in the weight of the hydrogel due to water or solution absorption, representing the ability of the adhesives to absorb water. In some situations, a large swelling ratio exerts pressure on surrounding brittle or sensitive tissues (Fig. 4B1). In contrast, in other conditions, a large swelling ratio is good for nutrient exchange and metabolic waste transfer (Fig. 4B2). Meanwhile, the swelling ratio of the adhesives is always calculated by the weight change of the adhesives in the medium compared with that of the initial weight and there are three kinds of initial weight as summarized in Fig. 4B3, which are dry weight obtained by freeze-drying or vacuum drying and initial wet weight. Since it is reported that vacuum drying will lead to a smaller swelling ratio than that resulting from the freeze-drying method, choosing the right initial weight according to how exactly the bioadhesives are applied is essential [141]. Regarding the degradation test, hydrolysis and enzymatic degradation are the two most common mechanisms (Fig. 4C). Synthetic materials have a higher likelihood of undergoing degradation through hydrolysis, whereas nature-based materials will mostly undergo enzymatic degradation.

The single most important factor before applying the bioadhesives is to make sure it can contact the living cells and tissues safely [142]. Six ways are commonly used to measure the cytotoxicity of bioadhesives (Fig. 4D1 to D6). 1. Prepolymer contents coincubation (Fig. 4D1): The prepolymer content is the polymer before forming the bioadhesives. There might be some uncrosslinked prepolymer during the application of bioadhesives, and this experiment is carried out to test the influence of these parts on the cells. 2. Leaching solution coincubation (Fig. 4D2): After the bioadhesives contact body liquids, the uncrosslinked/not fully-crosslinked parts will be released into the surrounding tissues, and the experiment can be used to evaluate their influences on biocompatibility. 3. Degradation products (Fig. 4D3): Degradation is very important for bioadhesives in certain applications, and coincubation of the degradation products of bioadhesives with cells can test their influences on cells after degradation. 4. Cell growth on the surface of the bioadhesives (Fig. 4D4). This is generally used when the bioadhesives are designed to support cell adhesion, migration and proliferation [143,144]. 5. Coincubation of bioadhesives with cells (Fig. 4D5): Not all the bioadhesives are suitable in this test and it is recommended that this method is mainly used for low-density materials. This is so because the materials will flow in the culture medium without pressing the cells [47,74,145,146]. In fact, this is also a good way to test the toxicity of the bioadhesives, which are not in one entire structure, like nanoparticles-based adhesives [146]. 6. Encapsulation of cells into the bioadhesives (Fig. 4D6): Bioadhesives are good vehicles for cell delivery [147]. For this purpose, to test their cytotoxicity, the cell encapsulation method is used. Additionally, it is also a good indicator of the ability of the bioadhesive to support the growth and differentiation of the cells inside the network. According to ISO 10993-5, more than 70% of cell viability is nontoxic. In papers, MTT, MTS, CCk-8, AlamarBlue®/PrestoBlue, photomicroscopy and Live&dead assays (Fig. 4E1 to E6) were used to assess the outcome of the testing. Among them, depending on the core molecules, MTT, MTS and CCK-8 are tetrazolium-based assays (Fig. 4E1 to E3) while AlamarBlue® and PrestoBlue® are tetrazolium-based (Fig. 4E4). Their concepts are that tetrazolium/resazurin compounds will be reduced because of the active metabolism of cells [148]. However, there is a chance that the bioadhesives can influence the result by interacting with the tetrazolium/resazurin compounds through non-enzymatic reduction, especially because the redox reaction is a commonly used method to fabricate bioadhesives in the DOPA system [149,150]. Since levels of DNA and RNA in cells are tightly regulated, the cellular nucleic acid content is a reasonable indicator of cell numbers [151]. Along with these, Picogreen® and CyQquant® detect cell viability through binding to the nucleic acid in the cells [152]. Those DNA-content-based methods might be a better choice for some redox reaction-based bioadhesives, but seldom of the biocompatibility of bioadhesives was seldom evaluated using those methods.

In summary, we have reviewed and categorized the different strategies used for cohesion. Bioadhesives might be one of the techniques that are most close to the market with serval available products and an increasing market. Most of the researchers focused on developing novel bioadhesion mechanisms, while ignoring cohesion mechanisms. A better way to facilitate commercialization might be to design the cohesion and adhesion mechanisms appropriately to the targeted application.
